# Antibiotic prophylaxis during dental implant surgery treatment in northwest China: a cross-sectional study

**DOI:** 10.3389/fpubh.2026.1746257

**Published:** 2026-05-29

**Authors:** Yao Lin, Jia Ju, Jing Huang, Jiao Yue, Lifei Cheng, Yanfei Ma, Wen Pan, Hongbo Wei, Bin Feng

**Affiliations:** 1 National Clinical Research Center for Oral Diseases, State Key Laboratory of Oral & Maxillofacial Reconstruction and Regeneration, Shaanxi Engineering Research Center for Dental Materials and Advanced Manufacture, Department of Pharmacy, School of Stomatology, The Fourth Military Medical University, Xi'an, China; 2 National Clinical Research Center for Oral Diseases, State Key Laboratory of Oral & Maxillofacial Reconstruction and Regeneration, Shaanxi Engineering Research Center for Dental Materials and Advanced Manufacture, Department of Oral Implants, School of Stomatology, The Fourth Military Medical University, Xi'an, China

**Keywords:** antibiotic prescription, bacterial resistance, dental implants, postoperative infection, prescribing patterns

## Abstract

**Background:**

This study was designed to investigate the existing status of antibiotic prophylaxis during dental implant surgery in northwest China.

**Methods:**

This cross-sectional study was conducted based on a web survey following the Strengthening the Reporting of Observational Studies in Epidemiology (STROBE) guidelines. The questionnaire comprised the basic information of respondents, the awareness of antibiotic prophylaxis, and the antimicrobial prescription habits of dentists performing dental implants. The questionnaire was sent individually to 2,099 dentists who performed dental implants in northwest China from April to December 2021. Data were collected and analyzed.

**Results:**

A total of 318 participants responded to the survey. Of the respondents, 65.41% (*n* = 208) routinely prescribed antibiotics during dental implant surgery, while only 0.94% (*n* = 3) did not prescribe them. A total of 141 (44.40%) respondents reported concerns regarding the indications for antibiotic use. The most frequently used antibiotics included penicillins, cephalosporins, and nitroimidazoles. Respondents were inclined to prolong antibiotics routinely for more than 24 h, specifically in patients with bone grafts, which reached 85.08%.

**Conclusion:**

Antibiotics are widely used to prevent infection during dental implant surgery in northwest China. Dentists are inclined to prolong the course of antibiotics routinely, both in healthy individuals and patients with bone grafts.

## 1 Introduction

Antimicrobial resistance (AMR) is a growing threat worldwide with considerable mortality implications. The Global burden of bacterial antimicrobial resistance in 2019 revealed that AMR was associated with an estimated 4.95 million deaths globally, with some authors suggesting that AMR could become the next pandemic if its drivers are not addressed (1, 2). The key driver of AMR is the inappropriate use of antibiotics, especially those from the Watch and Reserve groups with their increased resistance potential (3–6). These concerns led to the instigation of the WHO AWaRe (Access, Watch, Reserve) book to enhance future use of antibiotics—especially important given concerns with the robustness of antibiotic guidelines among low- and middle-income countries. The AWaRe book explicitly states that “Antibiotics are not needed before most dental procedures to prevent surgical site infections” (7, 8), highlighting the urgent need to reassess current antibiotic prophylaxis practices in dentistry.

Dental implant restoration is one of the best treatment options for restoring missing teeth due to positive long-term clinical outcomes (9, 10). However, dental implant failures occur, and bacterial contamination during implant surgery can lead to postoperative infections and early implant failures (11, 12). Dentists worldwide routinely use antibiotics to prevent infections during invasive dental procedures (13). Although several studies show that antibiotic prophylaxis is effective in preventing early implant failures, (11) evidence shows that antibiotic prophylaxis should not be used for “simple” implant procedures in systemically healthy individuals (14, 15). Some studies have examined the prescribing habits of dentists in different countries, including the Netherlands, Spain, Turkey, and Italy, to understand the use of antibiotics in implant surgery (16–19). The results show significant differences in the use of antibiotics in implant surgery.

Currently, there is no international consensus on antibiotic prophylaxis in implant surgery, which has led to large differences in the therapies prescribed by clinicians. Antibiotic prophylaxis during implant surgery is a controversial topic, and there are differing opinions on its necessity, (20, 21) so it is important to identify current prescribing habits during dental implant surgery.

China is among the largest manufacturers and consumers of antibiotics worldwide (22). The resistance rates for multiple pathogens have reached alarming levels (23). A national study, which included 48 healthcare facilities in six provinces in China, found that 52.9% of outpatient visits in primary care settings involved antibiotic use, with only 39.4% of outpatient antibiotic prescriptions deemed appropriate (24). This inappropriate use of antibiotics in primary care settings has contributed to the propagation of resistant organisms, presenting a major public health challenge. Given this context, the routine use of antibiotic prophylaxis for dental implant surgery in healthy patients becomes particularly questionable. Therefore, an action to promote the rational use of antibiotics in China is necessary. However, antibiotic use during dental implant surgery in China remains unclear.

The routine use of antibiotic prophylaxis for dental implant surgery remains controversial. Recent systematic reviews and meta-analyses have demonstrated that routine antibiotic prophylaxis may not be sufficiently effective to justify its use in straightforward implant procedures, and that antibiotics should not be used routinely in dental implant surger (25–28). Despite this, empirical practices and concerns regarding medical disputes contribute to antibiotic overuse in Chinese primary care settings. Dentists remain confused about the benefits of prophylaxis and desire clearer guidelines (13). Thus, research and education are urgently needed to transform prescribing behaviors. Nevertheless, the use of antibiotics during dental implant surgery in China requires more comprehensive investigation. The study aimed to investigate the current situation of antibiotic use and dentists' awareness in northwest China and to provide evidence-based data for developing guidelines for antibiotic prophylaxis during dental implant surgery.

A preprint has previously been published (29).

## 2 Materials and methods

This study obtained exemption from the ethics committee's review application (No. KQ-YJ-2023-077) because it did not perform any intervention in humans and did not use any personal data or biological samples of human origin. All collected data were completely anonymized. Informed consent was obtained for each questionnaire. This observational cross-sectional study followed the Strengthening the Reporting of Observational Studies in Epidemiology (STROBE) guidelines (30). All methods were performed following the relevant guidelines and regulations.

### 2.1 Participants

The Chinese Stomatological Association Oral Implantology Committee is China's largest and most authoritative academic organization for dental implantology. As of April 2021, 6,351 licensed dentists have registered as members, with membership requiring valid medical practitioner certification (http://www.cndent.com/). From April to December 2021, 2,099 dentist members in northwest China accepted to participate in the questionnaire survey circulated via WeChat. During this period, two reminders were sent to those who had not responded to the questionnaire. The hierarchical structure and certification requirements for dental clinical titles in China are detailed in Supplementary Table 1.

### 2.2 Study design

Based on the circumstances in China, the questionnaire, designed and produced by two experienced dental implantologists and pharmaceutical specialists, aimed to collect data concerning antibiotic prophylaxis prescription habits during dental implant surgery. The research team reviewed the questionnaire for intelligible and logical order. The question regarding the antibiotic regimen was based on published literature. To ensure the accuracy and consistency of all survey questions, a questionnaire prediction was first performed by 10 dentists. The questionnaire comprised three main parts, i.e., the basic information of respondents, the cognition degree, and the current use of antibiotic prophylaxis during dental implant surgery (Table 1).

**Table 1:** Questionnaire.

Questionnaire items	Response options
Part 1 basic information	
1. Your professional title	Junior (Resident physician)
	Intermediate (Attending physician)
	Senior (Deputy Chief Physician/Chief Physician)
2. Your educational background	Below Bachelor (Associate degree)
	Bachelor (Bachelor of Stomatology)
	Master (Master of Stomatological Medicine)
	Doctoral (Doctor of Stomatological Medicine)
3. Your majors	General stomatology
	Oral and maxillofacial surgery
	Oral implantology
	Prosthodontics
4. Type of your current place of employment	Stomatological hospital
	Department of Stomatology, general hospital
	Dental clinic
5. Number of implants performed per year?	≥100
	51–100
	≤ 50
Part 2 cognition of antibiotic prophylaxis during dental implant surgery	
6. Do you use antibiotics during the perioperative period of a dental implants?	Never
	Sometimes
	Always
7. During the perioperative period of dental implants, which problems do you think need to be provided necessary recommendations?	Indications for antibiotic use
	Antibiotic types
	Course of antibiotic use
	Starting time of antibiotic use
	Others
8. The reason and necessity of establishing the guideline for antibiotic prophylaxis during dental implant surgery	Reducing the overuse of antibiotics
	Standardizing the use of antibiotics
	Protect professional rights and interests in a lawsuit
	Others
Part 3 questions about antibiotic prescription habits during implant surgery	
9. When do you use antibiotics during a dental implant surgery?	Only preoperative
	Pre- and postoperative
	Only postoperative
	Only in special cases
10. If you suggest preoperative administration, then what is the specific time of administration?	More than 1 day prior
	1 day prior
	1 h prior
	Immediately
11. Antibiotic types: assuming that the patients has no antibiotic allergies, which antibiotic do you typically prescribe?(Multiple choice)	Penicillins (e.g., amoxicillin)
	Cephalosporins (e.g., cefazolin)
	Macrolides (e.g., roxithromycin)
	Lincomycin (e.g., clindamycin)
	Nitroimidazoles (e.g., metronidazole)
	Others
12. Course of antibiotics for dental implant surgery in healthy patients?	≤ 24 h
	>24 h
13. Course of antibiotics for dental implant surgery in patients with bone grafts?	≤ 24 h
	>24 h

### 2.3 Statistical methods

The SPSS version 22.0 (IBM Corporation, Armonk, NY, USA) software was used for data analysis. Measurement data were expressed as (±s). Students' *t*-test or analysis of variance (ANOVA) was used for comparison. Count data were assessed using proportions (percentages), and the comparison was conducted using the χ^2^ test. *P* < 0.05 indicated a statistically significant difference.

## 3 Results

### 3.1 Participants

A total of 318 participants completed and submitted their responses. The response rate was 15.15%.

### 3.2 Demographic data

All 318 respondents were licensed dentists. Among them, 27.99% held senior professional titles (chief physician or deputy chief physician), 50.94% held intermediate titles (attending physician), and 21.07% held junior titles (resident physician). Regarding education, 48.43% had bachelor's degrees, 27.04% master's degrees, and 11.32% doctoral degrees; 13.21% had education below bachelor's level. No dental technicians or other non-dentist professionals were included in the sample. 49.69% of the respondents performed more than 50 dental implant operations annually (Table 2).

**Table 2:** Basic information of the respondents (*n* = 318).

Basic information of respondents	Proportion	Habit of prescribing antibiotics (no., %)		
	(No., %)	Never	Sometimes	Always
Professional qualifications				
Junior (Resident physician)	67 (21.07)	1 (1.49)	26 (38.81)	40 (59.70)
Intermediate (Attending physician)	162 (50.94)	2 (1.23)	55 (33.95)	105 (64.82)
Senior (Deputy Chief Physician/Chief Physician)	89 (27.99)	0 (0)	26 (29.21)	63 (70.79)
Total	318 (100.00)	3 (0.94)	107 (33.65)	208 (65.41)
Education background				
Below Bachelor (Associate degree)	42 (13.21)	0 (0)	12 (28.57)	30 (71.43)
Bachelor (Bachelor of Stomatology)	154 (48.43)	1 (0.65)	52 (33.77)	101 (65.58)
Master (Master of Stomatological Medicine)	76 (27.04)	2 (2.32)	29 (33.72)	55 (63.95)
Doctoral (Doctor of Stomatological Medicine)	36 (11.32)	0 (0)	14 (38.89)	22 (61.11)
Total	318 (100.00)	3 (0.94)	107 (33.65)	208 (65.41)
Organization				
Stomatological hospital	92 (28.93)	3 (3.26)	30 (32.61)	59 (64.13)
Department of Stomatology general hospital	110 (34.59)	0 (0)	38 (34.55)	72 (65.45)
Dental clinic	116 (36.48)	0 (0)	39 (33.62)	77 (66.38)
Total	318 (100.00)	3 (0.94)	107 (33.65)	208 (65.41)
Professional types				
General stomatology	196 (61.64)	1 (0.51)	67 (34.18)	128 (65.31)
Oral and maxillofacial surgery	38 (11.95)	1 (2.63)	13 (34.21)	24 (63.16)
Oral implantology	51 (16.04)	0 (0)	10 (19.61)	41 (80.39)
Prosthodontics	33 (10.38)	1 (3.03)	17 (51.52)	15 (45.45)
Total	318 (100.00)	3 (0.94)	107 (33.65)	208 (65.41)
Number of implants placed per year				
≥100	88 (27.67)	0 (0)	26 (29.55)	62 (70.45)
51–100	70 (22.01)	1 (1.43)	19 (27.14)	50 (71.43)
≤ 50	16,050.31	2 (1.25)	62 (38.75)	96 (60.00)
Total	318 (100.00)	3 (0.94)	107 (33.65)	208 (65.41)

### 3.3 Current status of antibiotic prophylaxis in dental implant surgery

The results of our survey showed that 65.41% of dentists (*n* = 208) routinely prescribed antibiotics, 0.94% (*n* = 3) stated that they never prescribed any antibiotics and 33.65% (*n* = 107) respondents prescribed antibiotics when the patients had an underlying disease (Table 2).

### 3.4 Cognition of antibiotic prophylaxis

**Figure 1:**
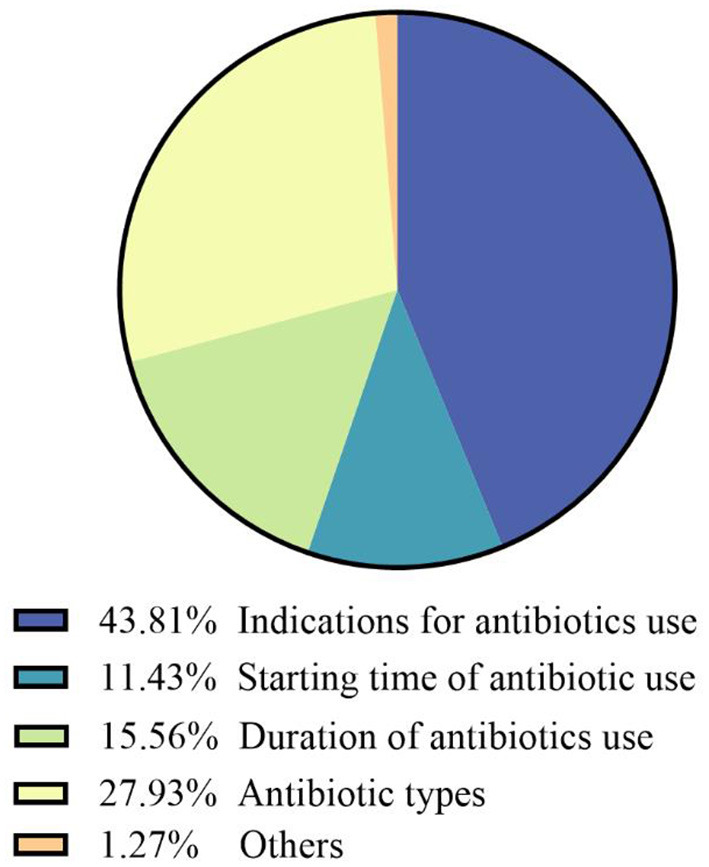
Questions about antibiotic use in dental implant surgery that the dentists desired to learn. This figure shows the questions dentists wanted to explore regarding antibiotic use in implant surgery, including indications, types, and duration of antibiotics.

The questionnaire included questions to survey the dentists' desire to learn about antibiotic use in implant surgery. A total of 43.81% of respondents wanted to know the indication of antibiotic use, 27.93% desired to know recommendations of antibiotic types, 15.56% about the duration of antibiotic use, and 11.43% about the initiation duration of antibiotic prophylaxis (Figure 1).

To establish guidelines for dental implant antibiotic prophylaxis, 77.36% of respondents considered that these could restrain doctors from prescribing more stringently, thus reducing the overuse of antibiotics and standardizing prescriptions among clinicians. 48.11% of respondents considered that these could protect practitioners in litigation cases, and 7.86% considered that these could reduce antimicrobial resistance.

### 3.5 Current status of antibiotic prophylaxis in dental implant surgery

#### 3.5.1 Timing of antibiotic prophylaxis

Except for three respondents who reported never prescribing antibiotics, 315 respondents prescribed antibiotics during implant surgery. Among them, 74.60% of respondents prescribed antibiotics pre-and post-operatively, 4.13% prescribed them only pre-operatively, and 15.87% prescribed antibiotics post-operatively (Table 3). 248 respondents who prescribed antibiotics pre-operatively during implant surgery advised their patients to start the regimen 1 h before surgery, accounting for 54.03 and 23.79% 1 day before the surgery (Table 4).

**Table 3:** Regimens and starting time of the medication (*n* = 315).

*n*/%	*n*	%	*n*	%
Category				
Only pre-operative				4.13%
More than 1 day prior	1	7.69%	13	
1 day prior	1	7.69%		
1 h prior	9	69.23%		
Immediately	2	15.38%		
Pre- and postoperative				74.60%
More than 1 day prior	39	16.60%	235	
1 day prior	58	24.68%		
1 h prior	125	53.19%		
Immediately	13	5.53%		
Only postoperative			50	15.87%
Only in special cases			17	5.40%
Total			315	100.00

**Table 4:** Antibiotics pre-operative during implant surgery.

*n*/%	*n*	%
Category		
More than 1 day prior	40	16.13%
1 day prior	59	23.79%
1 h prior	134	54.03%
Immediately	15	6.05%
Total	248	100%

#### 3.5.2 Type of antibiotics prescribed

**Figure 2:**
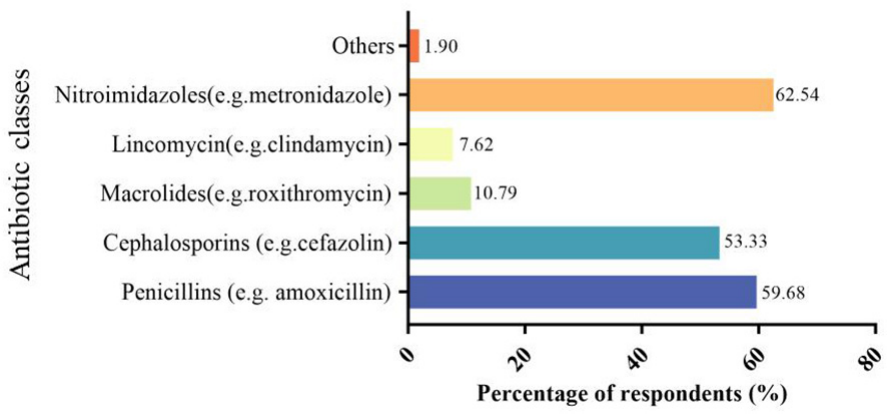
Proportion of antibiotic types. This figure displays the distribution of antibiotic types dentists prescribe during dental implant surgery.

**Figure 3:**
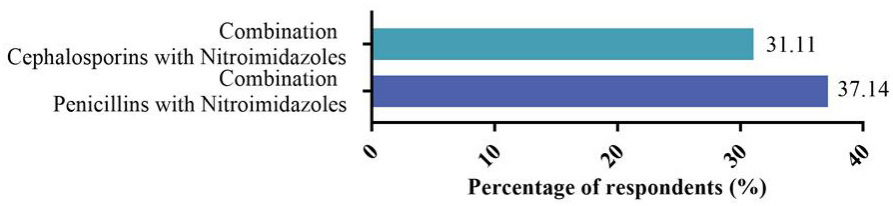
Combination of antibiotic types. This figure illustrates the various combinations of antibiotics, such as nitroimidazoles with penicillin or cephalosporins, used in dental implant surgeries.

The results of the questionnaire survey showed that nitroimidazoles, penicillins, and cephalosporins were the most commonly prescribed types of antibiotic prophylaxis in dental implant surgery (Figure 2). Considering the predominance of anaerobes in oral infections, respondents preferred the combination of nitroimidazoles. A total of 37.14% of respondents prescribed nitroimidazoles in combination with penicillin, and 31.11% prescribed nitroimidazoles in combination with cephalosporins (Figure 3). Nitroimidazoles accounted for the highest proportion (Figure 2).

#### 3.5.3 Regimens of antibiotic prophylaxis

According to the guideline specified in the Clinical Application of Antibiotics in China (2015), most maxillofacial surgeries are clean-contaminated surgeries, and the duration of antibiotic prophylaxis is less than 24 h. For healthy patients, 44.13% of respondents prescribed antibiotics for less than 24 h and 55.87% for more than 24 h. Among 55.87% of the respondents, those with primary, middle, and senior degrees accounted for 50, 53.13, and 65.17%, respectively. The respondents from the stomatological hospital, department of stomatology general hospital, and dental clinics accounted for 48.31, 56.26, and 61.21%, respectively (Table 5).

**Table 5:** Regimens of antibiotics prescribed for healthy patients during dental implant surgery (*n* = 315).

*n*/%	≤ 24 h/%		>24 h/%	
Category	*n*	%	*n*	%
Professional qualifications				
Junior (Resident physician)	33	50.00	33	50.00
Intermediate (Attending physician)	75	46.87	85	53.13
Senior (Deputy Chief Physician/Chief Physician)	31	34.83	58	65.17
Organization				
Stomatological hospital	46	51.69	43	48.31
Department of Stomatology general hospital	48	43.64	62	56.26
Dental clinic	45	38.79	71	61.21
Total	139	44.13	176	55.87

During dental implant surgery with bone grafts, 85.08% of respondents prescribed antibiotics for more than 24 h, and those with primary, middle, and senior degrees accounted for 81.82, 83.75, and 89.89%, respectively. Among the 85.08% of respondents, those from the stomatological hospital, department of stomatology general hospital, and dental clinics accounted for 83.15, 83.64, and 87.93%, respectively. The duration of antibiotics for patients with bone grafts was longer than that for healthy individuals (Table 6).

**Table 6:** Regimens of antibiotics prescription during dental implant surgery for patients with bone grafts (*n* = 315).

*n*/%	≤ 24 h/%		>24 h/%	
Category	*n*	%	*n*	%
Professional qualifications				
Junior (Resident physician)	12	18.18	54	81.82
Intermediate (Attending physician)	26	16.25	134	83.75
Senior (Deputy Chief Physician/Chief Physician)	9	10.11	80	89.89
Organization				
Stomatological hospital	15	16.85	74	83.15
Department of Stomatology general hospital	18	16.36	92	83.64
Dental clinic	14	12.07	102	87.93
Total	47	14.92	268	85.08

## 4 Discussion

This is a wide-ranging study concerning antibiotic prescriptions for dental implant surgery in China. All survey respondents were from northwest China, including stomatological hospitals, the Department of Stomatology in General Hospital, and dental clinics. Antibiotics are widely used during dental implant surgery in northwest China. Respondents prescribe their patients different drugs for different durations, with varying starting durations of antibiotic prophylaxis for the same implant surgery not only in China but also in other countries, including the Netherlands, (8) the UK, (5) Italy (11), and the U.S.A. (31). In China, the preferred antibiotic types in dental implant surgery were penicillins, cephalosporins, and nitroimidazoles. The duration of antibiotics during dental implant placement for patients with bone grafts usually exceeds 24 h. Among the respondents, 74.6% prescribed antibiotics both pre- and post-operative. Few respondents were aware of the evidence published to date, such as prescribing a single oral dose 1 h pre-operative (21, 32). Public awareness of antibiotic resistance needs to be improved (33). Without antibiotics, a surge in disputed cases of dental implants in China is likely to occur. Therefore, 48.11% of respondents believed that the development of guidelines could protect practitioners in litigation of medical disputes.

This finding is particularly concerning given the recent WHO AWaRe recommendations explicitly stating that antibiotics are not required before most dental procedures to prevent surgical site infections (7, 8). The considerable variation observed in our study, with 65.41% of dentists routinely prescribing antibiotics, suggests a significant gap between current practice in northwest China and global stewardship goals. The prescribing habits of antibiotics during implant surgery varied greatly across different countries. In Italy, (19) 84% of dentists in the Netherlands routinely prescribe antibiotics to prevent infection, but only 43.7% prescribe them (16). According to our survey, only 0.94% of people in China have never used antibiotics to prevent infection. In comparison, 3.3% in the Netherlands, (16) 13% in the UK, (13) and 25.9% in Santo Domingo, Dominican Republic (34) dentists performed implant surgery without using antibiotics. Although most dentists prescribe antibiotic prophylaxis, its protocols remain controversial for implant surgery (35). Several studies and reviews have indicated that antibiotic use had no efficacy in healthy individuals; on the contrary, it results in more adverse reactions and may lead to antibiotic resistance (36–38). Evidence has shown dental implants have a high success rate (39). The skill of dentists, the sterile environment of the surgical site, rigorous procedure, and the use of chlorhexidine pre- and post-operative are the important factors for the success of dental implant surgery (35). Recently, a consensus on preventive antibiotic therapy in dental implant procedures in Spain has recommended that no antibiotic prescription for patients be considered wrong (21). A growing body of evidence has shown the need for antibiotics use in special populations, such as patients with diabetes and those requiring bone grafts. Therefore, a more detailed questionnaire survey and professional analysis are needed in China to determine the use of antibiotics in oral implantology, which needs to be based on the patient's comorbidities and the difficulty of the surgery.

Our results showed that the common types of prophylactic antibiotics in dental implant surgery in China are penicillins, cephalosporins, and nitroimidazoles. Amoxicillin, amoxicillin-clavulanate, and metronidazole are the main types used in other countries (16, 19, 40, 41). According to the guidelines specified in China's Clinical Application of Antibiotics (2015), streptococcus and anaerobic bacteria are the main bacteria causing oral infections. The preferred treatment is using amoxicillin or amoxicillin clavulanic acid. For antibiotic types, the results reported were different from the recommendations in guidelines and evidence from the literature. This may be because approximately 10%−20% of patients stated an allergic reaction to penicillin. Many people have been considered “penicillin allergic” since their childhood in China. Therefore, dentists in China choose cephalosporins with a low incidence of allergic reactions. However, significant IgE-mediated or Tlymphocyte-mediated penicillin hypersensitivity is uncommon in clinical settings (42–44). Moreover, studies have shown that 80%−99% of these patients may no longer be considered allergic after allergy testing (45, 46). Most doctors do not question the possibility of incorrect labeling in China. Therefore, this questionnaire did not address the proportion of other antibiotic options for individuals with penicillin allergies, which is inaccurate. Accurate labeling of penicillin allergy in China is necessitated.

According to the guidelines in the Clinical Application of Antibiotics (2015) in China, oral surgery through oropharyngeal mucosa is a clean-contamination surgery. The antibiotic prophylaxis duration is usually less than 24 h. This survey showed that only 44.13% of respondents prescribed antibiotics for less than 24 h in healthy individuals, and 85.08% prescribed antibiotics for more than 24 h in patients with bone grafts. The respondents who prescribed antibiotics for more than 24 h had the highest qualifications. There was a significant correlation between the medical practitioner's qualification and the complexity of implant treatment, similar to the results of a Spanish study (47). The survey showed that over 80% of participants wanted standards established for antibiotic use in dental implant surgery. There are no references for the relationship between the difficulty of dental implant surgery and infection. It is unclear whether there is a need to distinguish between the use of antibiotics according to operation difficulty.

Our findings reveal a critical evidence-practice gap: 65.41% of dentists routinely prescribed antibiotics despite the WHO AWaRe book explicitly stating that antibiotics are not needed before most dental procedures (7, 8). Currently, China lacks specific national guidelines for antibiotic prophylaxis in dental implant surgery, forcing clinicians to rely on general antimicrobial guidelines that inadequately address procedure-specific nuances. National consensus guidelines are urgently needed to: (1) restrict routine antibiotic prophylaxis to high-risk cases (complex surgeries, immunocompromised patients); (2) standardize prescribing across clinical settings; (3) establish single-dose preoperative protocols when antibiotics are indicated; and (4) provide medico-legal clarity to address litigation concerns driving overuse. Recent evidence indicates that routine prophylaxis may not provide clinical benefit but increases adverse events, routine prophylaxis in healthy patients offers minimal clinical benefit (NNT >5) while contributing to adverse events and antimicrobial resistance (25, 48). With 80% of surveyed dentists desiring standardized protocols, the timing for guideline development is particularly opportune. We recommend that the Chinese Stomatological Association and National Health Commission convene expert panels to develop evidence-based guidelines aligned with international best practices, accompanied by targeted educational initiatives to transform prescribing behaviors. In conclusion, dual strategies are required: continued education to reduce overuse at the primary care level, and urgent establishment of national-level, procedure-specific guidelines to standardize practice.

This study has some limitations that warrant consideration. First, the questionnaire was limited, so it could not assess patients' physiological conditions. Second, cross-sectional studies cannot draw causal judgments. Prospective and systematic clinical studies are needed to determine the initiation time and antibiotic prophylaxis regimens in dental implant surgery. Fortunately, northwest China has a large population, and the questionnaire data are representative.

## 5 Conclusion

In summary, dentists widely use antibiotics to prevent infection during dental implant surgery in northwest China. However, there were several differences in the types, regimens, and antibiotic prophylaxis regimens. Therefore, it is urgent to issue relevant guidelines and establish a consensus to standardize antibiotic use in dental implant surgery to reduce overuse and prevent antibiotic resistance.

## Author's note

This article has been published in a preprint version. The link to the preprint is as follows: https://www.researchsquare.com/article/rs-3871632/latest.

## Data Availability

The original contributions presented in the study are included in the article/Supplementary material, further inquiries can be directed to the corresponding author.
