# Supplementary material for: Antibiotic prophylaxis during dental implant surgery treatment in northwest China: a cross-sectional study
Source: Front Public Health. 2026 May 29;14:1746257. doi: 10.3389/fpubh.2026.1746257 (PMC13260413; doi:10.3389/fpubh.2026.1746257)
Supplement: Supplementary file 1 [file Table_1.docx]

**Supplementary table 1. Hierarchical structure and certification requirements for dental clinical titles in China**

| Level | Chinese Title | International Equivalency: | Method | Focus |
| --- | --- | --- | --- | --- |
| Junior | Resident physician | House Officer / Junior Resident | Examination-based (national unified tests) | Basic competency, licensure |
| Intermediate | Attending physician | Staff Dentist / Registrar | Examination-based (national unified tests) | Clinical experience accumulation |
| Senior | Deputy Chief Physician/Chief Physician | Associate Professor /Professor of Clinical Dentistry | Review-based (peer evaluation + clinical performance metrics) | Clinical expertise, complex case handling, academic contribution |
